# Spectrum of opportunistic infections and associated factors among people living with HIV/AIDS in the era of highly active anti-retroviral treatment in Dawro Zone hospital: a retrospective study

**DOI:** 10.1186/s13104-018-3707-9

**Published:** 2018-08-20

**Authors:** Fithamlak Bistegen Solomon, Banchalem Nega Angore, Hailu Chare Koyra, Efrata Girma Tufa, Tezera Moshago Berheto, Mahlet Admasu

**Affiliations:** 1 Department of Medical Laboratory, Wolaita Sodo University, Wolaita Sodo, Ethiopia; 2 Department of Midwifery, College of Health Science and Medicine, Wolaita Sodo University, Wolaita Sodo, Ethiopia; 3 Department of Pharmacy, College of Health Sciences and Medicine, Wolaita Sodo University, Wolaita Sodo, Ethiopia; 4 Department of Human Nutrition and Reproductive Health, School of Public Health, Wolaita Sodo University, Wolaita Sodo, Ethiopia; 5 School of Medicine, Wolaita Sodo University, Wolaita Sodo, Ethiopia

**Keywords:** PLHIV, OI, Spectrum, Magnitude, Associated factors

## Abstract

**Objectives:**

The study aims to elucidate the spectrum, magnitude and determining factors of the major opportunistic infections in PLHIV patients currently receiving HAART.

**Results:**

A retrospective cross-sectional study was conducted at Tercha Hospital from 744 patient cards. The overall all prevalence of opportunistic infection was 658 (88.4%) developed OIs. Pulmonary tuberculosis, 118 (18%), severe community acquired pneumonia 107 (16.3%) and oral candidiasis 103 (15.6%) were the most common opportunistic infections. Disease stage [AOR = 3.22:95% CI 1.76–5.66], CD4 level [AOR = 2.53:95% CI 1.19–5.37], drug adherence [AOR = 3.02:95% CI 1.57–5.77] and hemoglobin [AOR = 2.49:95% CI 1.34–4.62] showed significant association with OIs. Higher magnitude of opportunistic infection with considerable proportion of AIDS defining illness was detected. So empowerment of skilled man power, health education and provision of antimicrobials is mandatory.

**Electronic supplementary material:**

The online version of this article (10.1186/s13104-018-3707-9) contains supplementary material, which is available to authorized users.

## Introduction

Human immunodeficiency virus (HIV), the causative agent of acquired immunodeficiency syndrome (AIDS) is the world’s most serious health and development challenge, affecting approximately 36.9 million people [1]. Ethiopia is one of the seriously HIV/AIDS affected countries in sub-Saharan Africa, with more than 1.3 million people living with the virus. Adult HIV prevalence in Ethiopia in 2016 was estimated to be 1.1% and 0.7% in Southern Nations, Nationalities and Peoples’ region (SNNPR) [2].

HIV became responsible for significant morbidity and mortality due to underlying immune-suppression which leads to life threatening opportunistic infections (OIs) during the natural course of the disease [3]. Scientific articles particulates, about 90% of HIV-related morbidity and mortality are caused by OIs compared to 7% due to opportunistic cancers and 3% due to other causes [4].

Since the advent of highly active antiretroviral treatment (HAART), the incidence of OIs in people living with HIV/AIDS, (PLHIV) reduced [5]. However, OI continue to cause morbidity and mortality in HIV/AIDS patients even after HAART hence an impediment to the attainment of the millennium development goals (MDGs) on health and poverty eradication particularly in resource poor countries is hard to achieve [6].

Opportunistic diseases like Candida esophagitis, Pneumocystis Carinii pneumonia (PCP), active pulmonary tuberculosis, Mycobacterium avium complex (MAC) infection, cytomegalovirus (CMV), Cryptococcal meningitis (CRM), Kaposis sarcoma (KS), and herpes zoster were prevalent in HIV patients [7]. In Ethiopian context, the most common opportunistic infections are oropharyngeal candidiasis, tuberculosis (TB), diseases of the central nervous system (CNS), sepsis, PCP, bacterial pneumonia, Kaposi’s sarcoma (KS), and lymphoma [8].

Opportunistic infections lead to frequent morbidity and mortality which shortens the life span of people with HIV infections and requires expensive treatments which becomes a burden for a developing country [9].

Timely intervention of OIs not only helps HIV positive persons to live longer but it also helps to prevent transmission spreading to others in the community [10]. Since pattern of infections vary from patient to patient and from country to country, this study aimed to elucidate the current frequency and spectrum of the major OIs/co-infections in HIV sero-positive individuals currently receiving HAART.

## Main text

### Methods

#### Study settings

The study was conducted at Tercha Referral Hospital situated at Tercha Town which is a capital city of Dawro Zone in South Ethiopia. It is serving a population of about 2 million people coming from different nearby zones and woredas. It also provides care and treatment for HIV/AIDS patients including ART (antiretroviral therapy), voluntary counseling test, provider initiative counseling and testing. The hospital provides inpatient and outpatient services including ART services. As of 2013, the Federal ministry of health, Ethiopia adopted the 2013 World Health Organization recommendation, that all HIV positives are eligible for ART. The ideal time for ART initiation depends on the clinical condition and readiness of the client. All PLHIV start ART as early as possible to all adults with a confirmed HIV diagnosis ready and willing regardless of their WHO clinical stages and CD4 counts [11].

#### Study design and period

Institution based cross-sectional study design was conducted from May 1 to August 30 2017.

#### Populations

All people living with HIV/AIDS (PLHIV) ≥ 18 years old currently using HAART.

#### Sample size determination

Nine hundred sixty-four patients were following their routine treatment in the hospital, of these 87 of them were children aged < 18 years. Thirteen of them were lost to follow up, 19 of them dropped from their course of treatment and 65 PLHIV were transferred to another health care facility. Incomplete data were obtained from 36 patients. Finally, 744 patients who are on ART follow up taking HAART from 2013 to 2017 were our sampling participants.

#### Operational definition

*Adherence* defined as good if adherence is > 95% (< 2 doses of 30 doses or < 3 dose of 60 dose is missed); poor if adherence is between 85 and 94% (3–5 doses of 30 doses or 3–9 dose of 60 dose is missed). Adherence was measured by pills count.

*Lost to follow up* Not seen since ≥ 1 month < 3 months.

*Dropped* Lost to follow up for > 3 months.

*SCAP* is defined is acute infection of the pulmonary parenchyma, and patients will often present with multiple organ failure with symptom onset in the community within 48 h of hospital admission.

*Transferred* A patient is referred to another health facility for care evidenced by his/her document.

#### Data collection procedures

Data were collected using a standard checklist prepared in English, retrieved from the hospital’s ART registry document which is a standard format for sending comprised data to health monitoring information system. Socio-demographic variables, clinical information, type of OIs, prophylaxis usage, WHO clinical staging, CD4 count, hemoglobin level, and weight were collected from patent charts by experienced nurses. Patients’ clinical records that were not complete or were missing data were omitted. The data collection format was checked for its completeness and consistency with the patient’s clinical records by a supervisor and the investigators on a daily basis. Five nurses working in other government health centers were involved in the data collection procedure.

#### Data processing and analysis

Data were entered and cleaned in Epi-info version 3.5 and analyzed using SPSS version 20. Bivariate analysis was conducted primarily to check association of each variable with OIs. Variables found to have association with the dependent variable at P-value of 0.2 in the bi-variate analysis was entered into multiple logistic regression model. The variables that showed significant association with P-value < 0.05 in the multivariate logistic regression was independent factors.

### Results

Out of 744 PLHIV, 443 (59.5%) were females and 254 (54.5%) were found in the age group of 18–30 years. Regarding to religion, majority of them 347 (46.6%), were orthodox. Among the participants, 587 (78.9%) of them were jobless and 432 (58.1%) were married. Urban dwellers in this study were 507 (68.1%). (Additional file 1: Table S1).

#### Clinical and lifestyle variables

Concerning exposure to social drugs 51 (6.8%) were using alcohol, 11 (1.4%) were smoking cigarette, and 8 (1.1%) were chewing kchat. Regarding to the current status of the patients, majority of them 597 (80.2%) were in WHO stage I. In case of CD4 count, 381 (51.2%) of them had CD4 count of < 200. Concerning hemoglobin, 182 (24.5%) of them having < 10 g/dl and 371 (49.8%) were having < 60 kg weight. Most of them 592 (79.6%) had not been using condom and 606 (81.4%) of the individuals had good HAART adherences (Table 1).

**Table 1:** Frequency distribution of health affecting behavior exposure, current health and adherence status of PLHIV (n = 744) in Tercha Hospital, 2017

Characteristics	Number	Percent (%)
Exposure to social drugs		
Alcohol	51	6.8
Cigarette	11	1.4
Kchat	8	1.1
No	675	90.7
WHO-stage		
T1	597	80.2
T2	48	6.5
T3	53	7.2
T4	45	6.1
Base line CD4 (cells/mm^3^)		
< 200	381	51.2
201–350	167	22.4
351–500	113	15.2
> 500	83	11.2
HGB		
< 10	182	24.5
≥ 10	562	75.5
Weight		
< 60	371	49.8
≥ 60	373	50.2
Condom use		
Yes	152	20.4
No	592	79.6
Adherence		
Good	606	81.4
Poor	138	18.6

#### Magnitude and spectrum of opportunistic infection

**Fig. 1:**
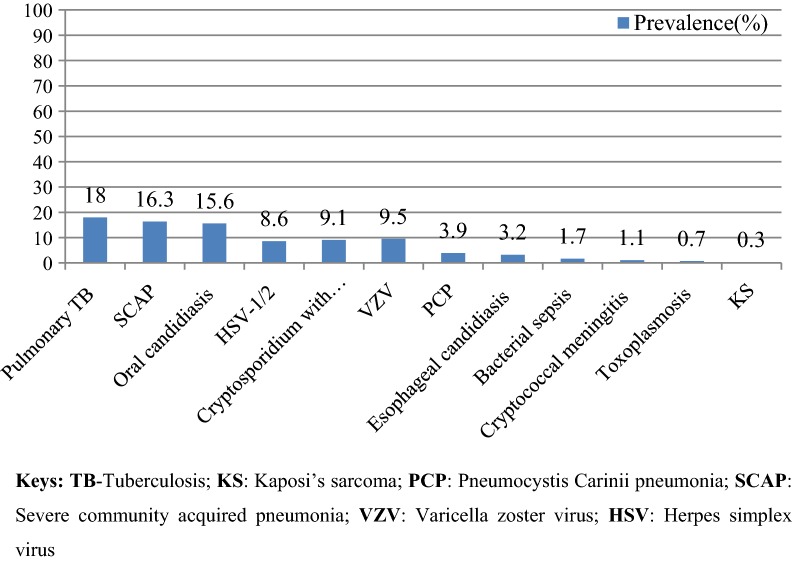
Spectrum of OIs among PLHIV in Tercha Hospital in 2017

Out of 744 individuals, 658 (88.4%) developed OIs, of which 232 (35.2%), 157 (23.9%), 146 (22.2%), and 123 (18.7%) developed 2, 3, ≥ 4, and 1 OI respectively. The spectrum of OIs was ranging from the common oral candidiasis to the life threatening CNS toxoplasmosis and the fungal cryptococcal meningitis. Pulmonary tuberculosis, 118 (18%) was the most common OI. Next to TB, Severe community acquired pneumonia (SCAP) 107 (16.3%) and oral candidacies 103 (15.6%) were the most common OIs in this study. Only five and 2 individuals were affected by Toxoplasmosis and KS respectively (Fig. 1).

#### Outcomes of OI

All individuals who develop OIs were treated for infections of which 107 (16.2%) of them died of the OI. The main cause of death were bacterial meningitis which accounts 16 (28.6%) and pulmonary TB and SCAP accounted for 13 (23.8%) of death each (Additional file 1: Table S2).

#### Factors associated with OI

Among the variables thought to have an association, WHO stage I, CD4 level, ART adherence and hemoglobin level showed significant association with OIs. PLHIV who are on WHO stage II–IV were 3 times more likely to develop OI than those who are on stage I [AOR = 3.22:95% CI 1.76–5.66]. Poor adherence became a predictor for developing OI in which the odds of developing OI among those individuals who have poor ART adherence was three times higher than those who have good adherence [AOR = 3.02:95% CI 1.57–5.77] (Table 2).

**Table 2:** Risk factors associated with OI among PLHIV in Tercha Hospital in 2017

Variable	OI		COR (95% CI)	P-value	AOR (95% CI)
	Yes	No			
Age					
≤ 40	307 (75.8%)	98 (24.2%)	1		1
> 40	251 (%)	88 (%)	1.74 (0.82–2.57)	0.28	2.31 (1.36–3.77)
Sex					
Male	273 (74%)	28 (26%)	1		1
Female	385 (86.9%)	58 (23.1%)	1.8 (0.69–4.28)	0.211	1.73 (.53–5.24)
Residence					
Urban	476 (93.95)	31 (6.1%)	1.42 (0.83–3.15)	0.096	2.15 (1.34–3.86)
Rural	182 (76.8%)	55 (33.2%)	1		1
Occupation					
Employed	131 (83.4%)	26 (16.6%)	1.39 (0.56–2.44)	0.26	2.35 (1.26–4.88)
Non-employed	527 (89.8%)	60 (10.2%)	1		1
Educational status					
No education	153 (95%)	8 (5%)	1		1
Primary	321 (89.9%)	36 (10.1%)	4. 5 (2.46–7.18)	0.48	0.46 (.014–7.68)
Secondary	157 (90.8%)	16 (9.2% %)	3.7 (1.13–8.2)	0.71	0.35 (.002–12.09)
Tertiary	27 (52.9%)	24 (47.1% %)	0.9 (0.17–3.1)	0.47	7.8 (.041–10.8)
Alcohol					
Yes	30 (58.8%)	21 (41.2%)	1		1
No	628 (90.3%)	65 (9.7%)	2.19 (.65–7.41)	0.24	0.61 (.087–3.78)
Cigarette smoking					
Yes	7 (63.6%)	4 (36.4%)	1.78 (1.07–2.96)	0.026	1.85 (1.06–3.24)
No	651 (88.8%)	82 (11.2%)	1		1
Kchat use					
Yes	4 (50%)	4 (50%)	1		1
No	654 (89%)	82 (11%)	4.13 (1.53–9.17)	0.54	1.76 (0.098–11.12)
WHO stage					
I	565 (94.6%)	32 (5.4%)	1		1
II–IV	93 (63.7%)	53 (36.3%)	2.56 (1.52–5.09)	***0.003***	3.22 (1.76–5.66)
CD4					
< 200	352 (92.9%)	29 (7.1%)	1		1
≥ 200	306 (84.3%)	57 (15.7%)	1.97 (1.06–3.64)	***0.021***	2.53 (1.19–5.37)
HGB					
< 10	165 (90.7%)	17 (9.3%)	1		1
≥ 10	493 (87.7%)	69 (12.3%)	2.01 (1.18–3.42)	***0.01***	2.49 (1.34–4.62)
Condom use					
Yes	103 (67.8%)	49 (32.2%)	1		1
No	545 (92.1%)	47 (7.9%)	1.72 (0.91–3.25)	0.096	2.12 (1.06–4.22)
Weight (kg)					
< 60	279 (75.2%)	92 (24.8%)	1		1
≥ 60	331 (88.7%)	42 (11.3%)	2.03 (0.95–4.33)	0.066	2.49 (1.02–6.12)
ART adherence					
Good	572 (94.4%)	34 (5.6%)	2.78 (1.52–5.09)	***0.001***	3.02 (1.57–5.77)
Poor	86 (62.3%)	52 (37.7%)	1		1

Bold italic values indicate significance of p value (p<0.05)

*TB* tuberculosis, *KS* Kaposi’s sarcoma, *PCP* Pneumocystis Carinii pneumonia, *SCAP* severe community acquired pneumonia, *VZV* Varicella zoster virus, *HSV* Herpes simplex virus

### Discussion

Magnitude of OIs in the current study, 88% was in harmony with 88.9% prevalence found in North western Ethiopia [12] but higher than the study conducted in the country as well as elsewhere ranging from 19.3 to 48% [12–16]. Variation across studies could be related to difference in duration of HAART, difference in CD4 level and difference in host immunity of study subjects.

Pulmonary tuberculosis was the most common leading OI in this study which was corroborated with findings reported in Ethiopia [12–14]. Similar evidences were also noticed in studies conducted worldwide [14, 17–19]. PTB is common among HIV-infected people even in high TB burden countries including Ethiopia.

Severe bacterial pneumonia was the next leading OI in the study 63 (25.5%) which is consistent with a study conducted in Cameroon 25% [20] and 31% in Uganda [21]. But much lower findings were also reported according to the national pneumonia prevalence of 5.2% [22] and 3.1% [12] in Debre Markos University. The higher finding in this study as compared with national study could be due to poor laboratory diagnosis in most part of the country where microbiological culture for the investigation for pneumonia is not under taken but the reverse is true in our setup.

Oral candidiasis prevalence (1.6%), which is the third most common OI in this study, corroborated with 16.4% prevalence rate in Zewditu memorial hospital [23]. In the contrary, it is the most common OI in Cambodia [24], and it is the second (5%) most common cause in Gondar [15]. Much higher, 7% in India [25] and 32%, Addis Ababa [26] and 21.6% Jimma [27] were also detected.

AIDS defining illness, like PCP, CRM, cryptosporidiosis and KS were identified in this study. Magnitude of PCP 3.9% observed in this study was higher than 2.1% in Zewditu memorial hospital [28], 1.5% prevalence in new Delhi [25] and 0.6% prevalence according the Ministry of health report from 1986 to 1990 [29] but much lower than 10% in Cambodia [24] and 12% in Brazil [30]. CRM prevalence 1.1% in this study was comparable with 1.2% in Addis Ababa [31] and 0.6% prevalence according to a study conducted in low and middle income countries [16]. But the current finding is lower than 2.5% reported in India [16], 5.9% in Tikur Anbesa Hospital [26], and 6.9% in Brazil [30].

Cryptosporidiosis finding of the present study 9.1% was corroborated with 13% prevalence detected in southern Ethiopia [32] but higher than 3.5% in Addis Ababa hospitals [23]. Difference in AIDS defining illness across studies could be due to difference in laboratory detection, CD4 level, and patient drug adherence.

Good HAART adherence became a predictor for the occurrence of OIs in this study which was corroborated with studies conducted in North western Ethiopia [12], Nigeria [17], France and other study conducted in sub Saharan African countries. This could be explained by the fact that good adherence will reduce viral replication and increase CD4 cells that intern decrease risk of new OI.

Human immunodeficiency virus infection is frequently associated with anemia where patients with more advanced HIV disease or a lower CD4 cell count had higher rates of anemia. Severe anemia was associated with a much faster rate of HIV disease progression and confirmed that anemia is a strong independent predictor of death. In different study settings, the prevalence of anemia has been estimated to be 30% in patients with asymptomatic HIV infection and 63–95% in persons with AIDS [33]. Hemoglobin showed significant association with spectrum of OIs in this study as it was evidenced in Debre Markos [12] where HIV Patients with a current hemoglobin level of < 10 mg/dl were more likely to develop OIs and similar findings were also noted in Nigeria [16].

CD4 count < 200/mm^3^ were found to be an independent predictor for developing OIs. This finding is in complete agreement with studies conducted in Gondar and India which reported high risk of developing OIs among this group [17–19]. This finding sounds true since CD4 cells play a central role in the activation of both humoral and cellular immunity to fight against infection.

Advanced baseline WHO stage was also another factor associated with OI in this study which was also observed in different studies conducted in the country and elsewhere [12, 16, 17, 34, 35].

## Conclusions

The overall prevalence of OI in the era of HAART is higher as compared with previous studies in the country. Significant level of AIDS defining illness was noticed. WHO stage II–IV, CD4 level, ART adherence and hemoglobin level became predictors for the occurrence of OIs. So skilled health professionals for proper diagnosis and management of OIs are mandatory and provision of adequate and sustainable antimicrobials and close monitoring and follow-ups are of critical importance.

## Limitation of the study

The retrospective nature of the study makes some the finding less informative and as a cross sectional study, cause-effect relationships cannot be assessed.

## Additional file

**Additional file 1: Table S1.** Frequency distribution of socio-demographic characteristics of PLHIV (n = 744) in Tercha Hospital in 2017. **Table S2.** Frequency distribution of outcomes of OIs of PLHIV in Tercha Hospital in 2017.

## Abbreviations

AOR: adjusted odds ratio
ART: antiretroviral treatment
CMV: Cytomegalo virus
COR: crude odds ratio
CRM: cryptococcal meningitis
AIDS: acquired immune-deficiency syndrome
KS: Kaposi’s sarcoma
HAART: highly active anti-retroviral treatment
HIV: human immunodeficiency virus
MAC: Mycobacterium avium complex
PLHIV: people living HIV/AIDS
OI: opportunistic infection
PCP: Pneumocystis Carinii pneumonia
PTB: pulmonary tuberculosis
SCAP: severe community acquired pneumonia
SPSS: statistical package for social sciences
WHO: World Health Organization
