# Supplementary material for: Spectrum of opportunistic infections and associated factors among people living with HIV/AIDS in the era of highly active anti-retroviral treatment in Dawro Zone hospital: a retrospective study
Source: BMC Res Notes. 2018 Aug 20;11:604. doi: 10.1186/s13104-018-3707-9 (PMC6102885; doi:10.1186/s13104-018-3707-9)
Supplement: Supplementary file 1 — Additional file 1: Table S1. Frequency distribution of socio-demographic characteristics of PLHIV (n = 744) in Tercha Hospital in 2017. Table S2. Frequency distribution of outcomes of OIs of PLHIV in Tercha Hospital in 2017. [file 13104_2018_3707_MOESM1_ESM.docx]

**Table S1: Frequency distribution of socio-demographic characteristics of PLHIV (n=744) in Tercha Hospital in 2017**

| Characteristics | | No | % |
| --- | --- | --- | --- |
| Sex | Male | 301 | 40.5 |
|  | Female | 443 | 59.5 |
| Age | 18-30 | 214 | 54.5 |
|  | 31-40 | 191 | 28.8 |
|  | 41-50 | 243 | 32.7 |
|  | >50 | 96 | 12.9 |
| Religion | Orthodox | 347 | 46.6 |
|  | Protestant | 343 | 46.1 |
|  | Muslim | 35 | 4.7 |
|  | Others | 19 | 2.6 |
| Occupation | Employed | 157 | 21.1 |
|  | Non employed | 587 | 78.9 |
| Marital status | Married | 432 | 58.1 |
|  | Widowed | 99 | 13.3 |
|  | Never-married | 101 | 13.6 |
|  | Divorced | 80 | 10.8 |
|  | Separated | 32 | 4.3 |
| Educational status | No education | 163 | 21.9 |
|  | Primary | 357 | 48 |
|  | Secondary | 173 | 23.3 |
|  | Tertiary | 51 | 6.8 |
| Place of residence | Rural | 237 | 31.9 |
|  | Urban | 507 | 68.1 |

**Table S2: Frequency distribution of outcomes of OIs of PLHIV in Tercha Hospital in 2017**

| Characteristics | | Number | Percent |
| --- | --- | --- | --- |
| Was the patient treated? | Yes | 658 | 100 |
|  | No | 0 | 0 |
| If yes, what was the outcome? | Improved | 490 | 74.5 |
|  | Not improved | 61 | 9.3 |
|  | Died | 107 | 16.2 |
| If died, was the exact cause identified? | Yes | 56 | 52.5 |
|  | No | 51 | 47.5 |
| Is the exact cause was identified, what was it? | Bacterial meningitis | 16 | 28.6 |
|  | Pulmonary TB | 13 | 23.8 |
|  | SCAP | 13 | 23.8 |
|  | Septic shock | 3 | 4.8 |
|  | Others | 11 | 19 |
